# Differences in buprenorphine prescribing readiness among primary care professionals with and without X-waiver training in the US

**DOI:** 10.1186/s12954-023-00918-3

**Published:** 2023-12-21

**Authors:** Berkeley Franz, Lindsay Y. Dhanani, O. Trent Hall, Daniel L. Brook, Janet E. Simon, William C. Miller

**Affiliations:** 1 Ohio University Heritage College of Osteopathic Medicine, Appalachian Institute to Advance Health Equity Science, Athens, OH USA; 2 Rutgers University School of Management and Labor Relations, Piscataway, NJ USA; 3 Department of Psychiatry and Behavioral Health, Ohio State University Wexner Medical Center, Columbus, OH USA; 4 Ohio State University College of Public Health, Columbus, OH USA; 5 Ohio University College of Health Sciences and Professions, Athens, OH USA; 6 Gillings School of Global Public Health, University of North Carolina Chapel Hill, Chapel Hill, NC USA

**Keywords:** Opioid-related disorders, Primary care, Misinformation, Buprenorphine, Addiction medicine, Medications for opioid use disorder

## Abstract

**Background:**

Medications for opioid use disorder (OUD) are effective at preventing overdose and infectious disease but are vastly under-prescribed in the US. For decades, prescribers faced additional training and regulation to prescribe buprenorphine which stigmatized the medication and lessened support for a harm reduction approach to treating opioid use disorder. The Drug Enforcement Administration removed the X-waiver requirement for prescribing buprenorphine in late 2022, which removed stigma and lessened important barriers to prescribing but also left training at the discretion of individual organizations. Our study aimed to assess differences in knowledge, confidence, and stigma regarding buprenorphine between those who went through the X-waiver training and those who did not, among practicing primary care providers (PCPs).

**Methods:**

We assessed buprenorphine prescribing readiness among primary care aligned outpatient providers in Ohio, USA. Using survey data, we conducted bivariate and regression analyses predicting primary prescribing outcomes. Primary outcomes measured knowledge of and confidence in buprenorphine, as well as perceived adequacy of one’s training. Secondary outcomes were attitudes toward patients with OUD, including bias toward OUD patients, stress when working with them, and empathy toward them. Participants (*n* = 403) included physicians, nurse practitioners, and physician assistants practicing in primary care aligned disciplines.

**Results:**

Survey data showed that PCPs who received X-waiver training were more likely to understand and have confidence in the mechanism of buprenorphine, and consider their training on treating OUD to be adequate. PCPs with an X-waiver showed more empathy, less negative bias, and experienced less stress when working with patients with OUD.

**Conclusion:**

Removing restrictive policies for prescribing buprenorphine is an important step to expanding access and reducing the stigma associated with opioid use disorder treatment. Yet, our findings suggest that the training received alongside regulation may be important for improving prescribing confidence and reducing stigma. Strategies to increase buprenorphine prescribing are unlikely to be effective without also expanding access to prescribing support for primary care providers across the career course.

## Introduction

Buprenorphine, one of the three medications for opioid use disorder (MOUD), prevents overdose in patients with opioid use disorder (OUD) and also reduces transmission of infectious disease [1–4]. Buprenorphine has similar efficacy to methadone, but unlike methadone which can only be dispensed through formal opioid treatment programs, it is available outside of hospital and formal substance use treatment settings in the US and therefore has the potential to increase access for patients [5]. Despite this potential, only an estimated 13% of patients with OUD receive medication [4], and MOUD prescribing is especially scarce in the primary care setting [6, 7]. Of particular concern is rural areas, where more than 50% of small and remote counties lacked a single MOUD provider in 2020 [8]. Office-based opioid treatment (OBOT), such as in the primary care setting, is more accessible for rural patients [9] and could increase access to evidence-based treatment in the absence of addiction medicine specialists and formal substance use treatment centers [10]. Experts have identified several barriers to MOUD prescribing, including lack of training, persistent stigma, and regulations at the federal, state, and organizational level [4, 11–13].

Buprenorphine is an essential part of a harm reduction approach to treating OUD. Harm reduction is defined broadly as efforts to minimize the harms associated with substance use [14]. Studies demonstrate that initiating buprenorphine often serves as a gateway to other needed health care for patients with OUD, such as preventive care and hepatitis C treatment [15–18]. Increasing access to buprenorphine in primary care is particularly important for harm reduction because this setting has lower access barriers and is a less stigmatizing care context than specialty or hospital settings [19]. Primary care providers are also experts in comprehensive care and chronic disease management [20], uniquely primed to treat conditions using the chronic care management (CCM) model [21], and thus are essential to normalizing OUD as a chronic disease and important public health concern [22, 23].

Despite the strong evidence for buprenorphine’s effectiveness and safety in the primary care setting, regulation of this medication varies across countries. In France, where the medication was first prescribed, buprenorphine is available in the primary care setting, and no additional training requirements are imposed for prescribers [24]. In other countries, however, buprenorphine is more heavily regulated, restricting the settings in which it is prescribed, imposing strict inclusion criteria for patients receiving the medication, limiting who can prescribe, and restricting the number of patients per prescriber [25].

In the US, trained and certified health-care providers have been able to use buprenorphine in outpatient care settings under the Drug Addiction Treatment Act since 2000. Prescribers were required to complete training, file a formal application (adding an X to their DEA number, known as an “X-waiver”), and submit to periodic audits to prevent medication diversion [26]. In 2017, this waiver was expanded to include nurse practitioners and physician assistants, with additional training hours required for advanced practice providers (APPs) [27]. Critics of the X-waiver argued that removing regulation of buprenorphine prescribing was essential to improving access and decreasing the stigma around MOUD [12]. Requiring extra steps to prescribe buprenorphine, when other opioid medications were not similarly regulated, created substantial access challenges for patients with OUD and furthered hesitance to prescribe the medication [12]. Amid growing pressure to adopt a harm reduction approach to addressing the US opioid epidemic, the regulation of buprenorphine was gradually weakened. In 2021, training requirements were removed for physicians although the X-waiver remained in place [28]. In late 2022, the requirement to hold an X-waiver to prescribe buprenorphine was removed altogether through an executive order passed by President Biden [7].

Despite the clear benefits of removing federal buprenorphine regulations, we do not yet have data on what impact these changes will have on buprenorphine prescribing and expanding access to care. Given that just 3.6% of family physicians had received an X-waiver in 2015 [29], and just half of prescribers with an X-waiver ever prescribed the medication [30], it is unlikely that policy changes alone will be enough to meet patient needs for medication [31]. Absent federal regulation, there is also an urgent need to establish new forms of prescribing support to increase confidence and readiness to prescribe the medication. Existing evidence suggests that health-care professionals who received the X-waiver had improved knowledge following training [32], but it is not clear if this training also bolstered confidence using the medication and lessened stigma toward patients with OUD.

The amount of training on buprenorphine prescribing in health professions training currently varies widely [33], and there are opportunities to improve confidence in prescribing buprenorphine among new health-care professionals and those already in practice, such as through Project ECHO or other virtual mentoring initiatives [34, 35]. Despite the longstanding requirements for health-care professionals (HCPs) to complete buprenorphine training prior to prescribing to patients with OUD, we do not know how effective additional training on buprenorphine prescribing was, or whether individuals who received the training systematically differed from those who did not complete additional training. These findings are an important step toward developing prescribing support for primary care providers to help close the gap in buprenorphine access in the US.

The aim of this study was to assess differences in medication knowledge, stigma, and beliefs about buprenorphine among primary care providers with and without DEA X-waivers. This study is significant as it presents the last available data on PCPs prior to the removal of X-waiver requirements and assesses both knowledge and confidence as well as atittudes toward patients with OUD. That is, the current study builds on prior work documenting the effects of the X-waiver to also link the obtainment of the X-waiver to attitudes toward patients with OUD, which we argue that it is integral to consider as these attitudes are an important barrier to closing the access gap to evidence-based MOUD. We considered whether receipt of an X-waiver was associated with knowledge and confidence in buprenorphine, bias and empathy toward patients with OUD, and stress experienced when working with this patient population. These findings will inform future efforts to establish buprenorphine prescribing support to expand confidence and willingness to use these medications in the primary care setting.

## Methods

### Study population

We surveyed 403 PCPs licenced to practice in Ohio. We focused on a single state because we wanted to understand geographic differences in prescribing attitudes and behaviors across the state, and because several US states, including Ohio, have separate statewide regulation of buprenorphine prescribing [36]. The broader study was focused on understanding willingness to use buprenorphine in the treatment of OUD, with the long-term endpoint of developing a prototype prescribing support intervention to increase buprenorphine prescribing in rural primary care practice. Participants were eligible to participate if they were a health-care professional eligible to prescribe medications such as MOUD. We included physicians, nurse practitioners (NPs), and physician assistants (PAs) who were practicing in one of the following primary care aligned disciplines: family medicine, internal medicine, obstetrics/gynecology, and emergency medicine. We also included three additional disciplines that have a high likelihood of interacting with patients with OUD: addiction medicine, infectious disease, and psychiatry.

Using G*Power, we set an a priori goal of 400 participants to adequately power our analyses predicting primary prescribing outcomes and secondary attitudinal outcomes. We recruited participants through multiple mechanisms to increase participation and diversity in our sample. First, we emailed 20,143 potential participants using contact information available in the State Board of Medical and Nursing Licensing rosters. Second, we advertised the study through newsletters and listservs in partnership with several professional associations in Ohio. Finally, we worked with health professions training programs in the state to share the survey invitation with alumni.

We cannot calculate a response rate, given that state licensure rosters contain many out-of-date email addresses (e.g., email addresses associated with the university where individuals trained), and because we utilized newsletters and listservs associated with professional organizations and health professions schools. We sent two reminder emails approximately 3 and 7 days after the original email invitation. The percentage of emails that were opened ranged from 31.2 to 60.2%. Our survey was in the field for 2.5 months in the fall of 2022, closing in late December. A total of 659 participants began the survey of which 545 met eligibility criteria. Most participants with missing data exited the survey immediately after entering, and a total of 403 participants completed all questions. Participants who completed the survey received a $20 gift card as compensation. We utilized a second survey to collect identifying information, so that participants remained anonymous. The study was approved by the [name redacted] internal review board, and all respondents provided electronic informed consent prior to participation.

### Data and measures

Survey measures included closed-ended questions on the mechanisms of MOUD, beliefs about MOUD effectiveness, attitudes toward patients with OUD, practice characteristics, including receipt of the X-waiver and panel size, and demographic characteristics. Our primary outcomes measured knowledge of and confidence in buprenorphine, as well as perceived adequacy of one’s training. Because limited measures existed in the literature for these constructs, we developed primary outcomes using research team consensus. This team included two social scientists, an infectious disease physician and epidemiologist, an addiction medicine physician, and a biostatistician. Perceived effectiveness of buprenorphine was measured using a single item which asked: “How effective is buprenorphine at preventing overdose deaths?” Perceived likelihood of remission was measured with a single item which asked: “How likely are patients to enter remission from opioid use disorder with buprenorphine alone?” Both were measured on a 5-point response scale ranging from (1) strongly disagree to (5) strongly agree. Knowledge of buprenorphine was measured with a multiple-choice, board-style question asking participants to identify the correct mechanism of action for buprenorphine. Distractors included mechanisms of action from other MOUD and naloxone. Training was measured with a single item asking: “I feel that I have received adequate training for treating patients with opioid use disorder,” with 5-point response scale ranging from (1) strongly disagree to (5) strongly agree.

Secondary outcomes were attitudes toward patients with OUD, including explicit bias toward patients with OUD, stress experienced when working with patients with OUD, and empathy toward patients with OUD. Bias was measured using a previously validated 8-item measure [37] with five response options ranging from (1) strongly disagree to (5) strongly agree, which showed strong internal consistency using Cronbach’s alpha (*α* = 0.80). How often participants experienced stress when working with patients with OUD was a 2-item measure (*α* = 0.86); and five response options ranged from (1) never to (5) every day. We adapted a previously validated empathy scale to measure empathy toward patients with OUD. This 6-item measure (*α* = 0.93) asked participants to indicate the extent to which they felt various responses (e.g., sympathetic and warm) toward patients with OUD, on a 5-point response scale ranging from (1) not at all to (5) extremely. Our focal independent variable was whether participants had an active DEA X-waiver, using a binary Yes/No variable. We measured provider demographics, including sex, age, race, and training credential (physician, NP, or PA), whether the participant was board-certified in family medicine (Yes/No), whether the participant practiced in family medicine as compared to other specialties, and the average number of hours worked per week.

### Analysis

To assess the relationship between holding an X-waiver and our primary and secondary outcomes, we first calculated descriptive statistics to characterize the sample of participants with and without X-waivers. We next conducted independent samples t-tests to compare mean scores for participants with and without X-waivers on each of our continuous dependent variables and Chi-square analyses to compare counts for participants with and without X-waivers on each of the dichotomous outcome variables. Alpha level was set to 0.05. We finally computed regression models for each primary and secondary outcome variable to assess whether the relationship between holding an X-waiver and our outcomes of interest held after accounting for other known predictors. Each regression model controlled for provider demographics. All statistical analyses were conducted using Stata 15 [38].

## Results

### Descriptive

Among our sample, 30.0% (*n* = 121) were NPs, 43.1% (*n* = 174) worked as physicians, and 27.0% (*n* = 109) were PAs (Exhibit 1). For area of practice, 54.3% (*n* = 285) of participants worked in family medicine, with the remaining participants distributed across internal medicine (17.5%, *n* = 92), emergency medicine (16.8%, *n* = 88), addiction medicine (10.7%, *n* = 56), psychiatry (5.5%, *n* = 29), obstetrics/gynecology (4.6%, *n* = 24), infectious disease (2.9%, *n* = 15), and pain medicine (1%, *n* = 5). Approximately 60.6% (n = 245) were female, and the average age of respondents was 42.3 years old (SD = 12.0). Participants had been in practice an average of 17.8 years (SD = 11.6) and worked an average of 41.3 h per week (SD = 13.1). Approximately 47.6% (*n* = 247) of participants held an X-waiver at the time of the study.

### Bivariate analysis

For all four primary training outcomes, participants who held X-waivers scored significantly different than those without this training. Participants with an X-waiver were more likely to believe that buprenorphine was effective at reducing withdrawal symptoms (*t*_(407)_ = −  7.3, *p* < 0.001, *g* = − 0.72). Results indicated that 55.1% (*n* = 113) of X-waiver recipients believed buprenorphine to be very or extremely effective, as compared to 22.6% (*n* = 46) of PCPs without an X-waiver. The results were similar for believing that patients with OUD are likely to recover with buprenorphine alone (*t*_(407)_ = −  4.8, *p* < 0.001, *g* = − 0.47); 66.8% (*n* = 137) of participants with X-waivers believed that patients with OUD are either somewhat or extremely likely to enter remission with buprenorphine, as compared to 47.1% (*n* = 96) of their counterparts. Moreover, 71.6% (*n* = 151) of participants with an X-waiver could correctly identify the mechanism of buprenorphine as compared to 49.1% (*n* = 105) of individuals without an X-waiver (*Χ*^2^_(424)=_22.5, *p* < 0.001, *φ* = 0.23). Participants with X-waivers were more likely to report that they had received adequate training for treating OUD (*t*_(440)_ = −  10.6, *p* < 0.001, *g* = − 1.01); 71.9% (*n* = 156) of PCPs with an X-waiver perceived their training to be adequate, compared to 28.0% (*n* = 63) of PCPs without an X-waiver.

Across our secondary outcomes, participants with X-waivers were also significantly different than their counterparts. Explicit bias toward patients with OUD was lower among individuals with X-waivers (*t*_(402)_ = 6.1, *p* < 0.001, *g* = 0.61). The average bias score among participants with X-waivers was 1.8 (SD = 0.60) as compared to 2.2 (SD = 0.70) among participants without this training. Participants with X-waivers also reported less frequent stress when working with patients with OUD (*t*_(443)_ = 4.7, *p* < 0.001, *g* = 0.44); 32.0% (*n* = 70) of participants with X-waivers reported commonly experiencing stress when caring for patients with OUD, as compared to 49.3% (*n* = 112) of their counterparts. Participants with X-waivers also reported greater empathy for patients with OUD (*t*_(403)_ = −  5.5, *p* < 0.001, *g* = − 0.54), with 27.7% (*n* = 56) vs. 16.8% (*n* = 34) of their counterparts identifying as “quite a bit” or “extremely” empathetic toward patients with OUD (Table 1).

**Table 1:** Descriptive and bivariate statistics by X-waiver status

Variable	X-waivered (*n* = 202)	Non-X-waivered (*n* = 201)	*P* value	Scale or range	
				Min	Max
Family medicine	42.6	**57.3**	.012	0	1
Physician assistant	50.5	49.5	.911	0	1
Nurse practitioner	54.5	45.5	.232	0	1
Physician	46.6	53.5	.228	0	1
Age	**43.7**	41.1	.029	25	74
Years in job	17.6	17.9	.771	1	35
Work hours	41.6	41.0	.672	0	84
Female	46.5	53.5	.083	1	2
Buprenorphine recovery	**3.6**	3.1	< .001	1	5
Buprenorphine effectiveness	**3.6**	2.9	< .001	1	5
Adequate training	**3.8**	2.6	< .001	1	5
Buprenorphine knowledge	**59.0**	41.0	< .001	0	1
Stress treating OUD	3.1	**3.6**	< .001	1	6
Empathy	**3.4**	2.9	< .001	1	5
Explicit bias	1.8	**2.2**	< .001	1	2

Bold text indicates significance at *p* < .05

*M* Mean, *SD* Standard deviation, *Min* Minimal value, and *Max* Maximum value

### Regression analysis

After accounting for individual demographic factors and practice characteristics, receipt of an X-waiver remained a significant predictor of all four primary outcomes (Table 2). Holding an X-waiver was associated with a 0.66-point increase in believing buprenorphine which was effective at reducing withdrawal symptoms (*p* < 0.001); a 0.26-point increase in believing buprenorphine alone is effective at helping patients with OUD experience remission (*p* < 0.001); a 1.2-point increase in perceived training adequacy (*p* < 0.001); and a 23% greater likelihood of correctly identifying the mechanism of buprenorphine (*p* < 0.001). Among secondary outcomes (Table 3), holding an X-waiver was associated with a 0.45-point decrease in bias toward patients with OUD (*p* < 0.001), a 0.46-point decrease in perceived stress when working with patients with OUD (*p* < 0.001), and a 0.51-point increase in empathy toward patients with OUD (*p* < 0.001).

**Table 2:** Regression coefficients for the relationship between receipt of X-waiver and primary outcomes (*n* = 403)

	Likelihood of believing buprenorphine is effective			Likelihood of believing buprenorphine helps people enter remission			Training			Predicting buprenorphine knowledge		
	Coef.	SE	95% CI	Coef.	SE	95% CI	Coef.	SE	95% CI	OR	SE	95% CI
*Unadjusted*												
X-waiver	0.66^±±^	.09	0.49, 0.84	0.47^±±^	0.10	0.28–0.67	1.21^±±^	0.11	0.99, 1.44	2.61^±±^	0.54	1.75, 3.90
*Adjusted*												
X-waiver	0.66^±±^	0.09	0.47, 0.84	0.48^±±^	0.10	0.28, 0.68	1.18^±±^	0.12	0.94, 1.42	2.75^±±^	0.60	1.79, 4.22
Female	-0.18	0.11	− 0.39, 0.03	0.15	0.11	− 0.08, 0.37	− 0.25	0.14	− 0.52, 0.03	1.04	0.26	0.64, 1.70
Age	− 0.00	0.00	− 0.01, 0.01	− 0.01	0.00	− 0.02, 0.00	0.00	0.00	− 0.01, 0.02	0.99	0.01	0.97, 1.01
Work hours	0.01*	0.00	0.00, 0.01	0.00	0.00	− 0.00, 0.01	0.00	1.16	− 0.01, 0.01	1.01	0.01	0.99, 1.03
Years in job	0.00	0.00	− 0.00, 0.01	− 0.00	0.00	− 0.01, 0.01	0.00	0.01	− 0.01, 0.01	0.99	0.01	0.97, 1.01
Physician assistant	− 0.13	0.12	− 0.37, 0.12	− 0.19	0.13	− 0.45, 0.08	0.23	0.16	− 0.09, 0.56	0.49*	0.14	0.28, 0.87
Nurse practitioner	0.15	0.13	− 0.10, 0.40	− 0.32*	0.14	− 0.59, − 0.05	− 0.13	0.17	− 0.46, 0.20	0.56	0.17	0.31, 1.01
Physician	REF	REF	REF	REF	REF	REF	REF	REF	REF	REF	REF	REF
Family medicine	− 0.03	0.11	− 0.24, 0.18	0.06	0.12	− 0.17, 0.28	0.21	0.14	− 0.07, 0.49	0.79	0.20	0.48, 1.31

^*^p < .05; ^±^*p* < .01; and ^±±^*p* < .001

*REF* Reference group, *Coef.* Coefficient, *OR* Odds ratio, *SE* Standard error, and *CI* Confidence interval

**Table 3:** Regression coefficients for the relationship between receipt of X-waiver and secondary outcomes (*n* = 403)

	Empathy			Stress			Bias		
	Coef.	SE	95% CI	Coef.	SE	95% CI	Coef.	SE	95% CI
*Unadjusted*									
X-waiver	0.50 ± ±	0.09	0.32, 0.68	− 0.49 ± ±	0.09	0.48, 0.84	− 0.41 ± ±	0.07	− 0.54, − 0.28
*Adjusted*									
X-waiver	0.51 ± ±	0.09	0.33, 0.69	− 0.46 ± ±	0.11	− 0.67, − 0.24	− 0.43 ± ±	0.07	− 0.56, − 0.30
Female	0.14	0.11	− 0.07, 0.35	− 0.27*	0.13	− 0.52, − 0.02	− 0.29 ± ±	0.08	− 0.44, − 0.14
Age	− 0.00	0.00	− 0.01, 0.01	− 0.00	0.00	− 0.01, 0.01	0.00	0.00	− 0.01, 0.01
Work hours	0.00	0.00	− 0.00, 0.01	0.00	0.00	− 0.02, 0.01	− 0.01*	0.00	− 0.01, 0.01
Years in job	− 0.01*	0.00	− 0.02, − 0.00	− 0.00	0.00	− 0.01, 0.01	0.00	0.00	− 0.00, 0.01
Physician assistant	0.01	0.12	− 0.24, 0.25	− 0.03	0.15	− 0.32, 0.26	− 0.00	0.09	− 0.18, 0.17
Nurse practitioner	− 0.01	0.13	− 0.26, 0.24	0.04	0.15	− 0.26, 0.34	0.06	0.09	− 0.13, 0.24
Physician	REF	REF	REF	REF	REF	REF	REF	REF	REF
Family medicine	0.01	0.11	− 0.20, 0.22	− 0.11	0.13	− 0.36, 0.14	− 0.00	0.08	− 0.15, 0.15

^*^p < .05; ^±^*p* < .01; and ^±±^*p* < .001

*REF* Reference group, *Coef.* Coefficient, *OR* Odds ratio, *SE* Standard error, and *CI* Confidence interval

## Discussion

Our findings suggest that individuals who completed X-waiver training have greater knowledge of buprenorphine, greater confidence in this medication, and are more likely to perceive their training on OUD to be adequate. Receipt of an X-waiver was also associated with our secondary outcomes measuring attitudes toward patients with OUD, including greater empathy toward patients with OUD, feeling less stressed when working with patients with OUD, and lower explicit bias toward this patient population. Although statistically significant, the magnitude of these differences was smaller than those observed for knowledge and perceived training adequacy.

There are two primary explanations for these findings: 1) The training made a difference; or 2) people who sought training already had less bias/more knowledge, which motivated them to get a waiver. Although neither explanation is exclusive, the possibility that training improved attitudes and knowledge makes conceptual sense, given existing studies which have found statistically significant improvement in knowledge and trainee satisfaction after X-waiver training [39]. One qualitative study of X-waiver recipients found that the vast majority were compelled to take the training as part of educational or employment requirements, rather than because of their own interest, providing support for the first explanation [11]. Although our cross-sectional data do not allow us to determine whether the waiver training itself was responsible for these group differences, we are encouraged by the findings that individuals who received the training subjectively assessed their training level as adequate for treating OUD.

X-waiver training was associated with improvements in knowledge and confidence in one’s training, more so than improvements in attitudes toward buprenorphine or toward patients with OUD. These findings are important because they suggest that knowledge-focused training is necessary but may not be sufficient to drive prescribing increases among PCPs. These findings offer a key contribution to the literature as they extend beyond previous studies which only measured improved knowledge associated with receipt of the X-waiver [32]. It is important to also consider the association between obtaining the X-waiver and attitudes toward patients because prior studies document that attitudinal barriers can significantly limit treatment access [10]. Other barriers to prescribing, such as confidence in the effectiveness of buprenorphine and stigma toward patients with OUD [40], should be specifically targeted in the future prescribing support interventions.

Beyond provider-level barriers, it is important to note that there are patient-, pharmacy-, and organizational-level barriers that remain and which may be exacerbated in some settings, including rural communities [41]. Patients, for example, may also hold negative attitudes toward MOUD as the medications themselves have been stigmatized [42, 43]. Buprenorphine prescriptions also require available pharmacies to fill the medication, and existing studies suggest that pharmacists may be hesitant to dispense the medication [44, 45]. Finally, organizational policies and stigma may limit buprenorphine prescribing regardless of HCP training and attitudes [46, 47]. Implementation strategies to address barriers across multiple settings [48] are necessary to expand access to buprenorphine.

### Implications for policy and practice

Training for substance use disorders, including OUD, is minimal in health professions education and varies greatly across institutions and residency training programs [33, 49]. Although buprenorphine regulations limited access to this medication and contributed to the substantial treatment gap that exists currently, the requirements to receive training on MOUD may have been helpful for increasing knowledge and confidence in buprenorphine. By removing the training requirement along with deregulation, many people may not receive or seek training, which could affect their willingness and ability to prescribe confidently.

With training requirements no longer in place, these findings provide support for expanding access to buprenorphine training, which may not only improve knowledge of this medication but help increase willingness and confidence in treating patients with OUD. Several policy proposals have been introduced which have the potential to fill the current training gap [12, 50]. Our study suggests that a key priority should be expanding the focus of training beyond MOUD knowledge to include stigma reduction and efforts to increase confidence in using buprenorphine in primary care practice. Standardizing training in medical and health professions education, as well as residency training, is critical to expanding the workforce [33, 49]. At the same time, PCPs recently entering practice are the most likely to prescribe buprenorphine [31], suggesting that we must better support clinicians already in practice. To accomplish this, priority should be given to making training convenient and free to PCPs in practice. Training should also be tailored to the primary care setting, given the unique practice environment and the expertise PCPs have in managing chronic disease. Finally, we must move beyond thinking of training as a static event. Although a single training event, like the X-waiver, has shown success for increasing knowledge of MOUD [39] mentorship programs, or long-term prescribing support may be even more likely to instill the confidence necessary to sustainably integrate MOUD prescribing into primary care practice.

### Limitations

Study findings should be weighed against important limitations in our sample and study design. We did not employ a probability-based sampling method because of the challenges in enrolling practicing clinicians into research studies. Our sample is not representative of health-care professionals in Ohio or nationally, and the findings may not be generalizable. To address this limitation, we recruited through several mechanisms to increase opportunities to participate in the study and improve sample diversity. There are several potential confounds that were not measured in the study, including how long participants held the X-waiver, and whether participants had a personal or family experience with SUD. Future studies should include these measures and determine if the relationship between X-waiver training and both primary and secondary outcomes remains.

Our data are also limited by the cross-sectional design used to measure receipt of the X-waiver and both primary and secondary outcomes. It is possible that individuals with greater confidence in buprenorphine and more positive feelings toward patients with OUD were more inclined to enroll in the X-waiver training. Future randomized controlled trials are needed to determine if training and other long-term forms of prescribing support meaningfully improve prescribing outcomes among providers, alongside attitudes toward MOUD and patients with OUD.

## Conclusion

Participants who received an X-waiver were significantly different than their counterparts in knowledge of buprenorphine, confidence in the medication, and in attitudes toward treating patients with OUD. Removing the X-waiver regulation was an important step forward in destigmatizing buprenorphine. Nonetheless, the X-waiver did constitute additional training in addiction medicine, which is critically needed among primary care providers. Efforts to expand access to buprenorphine in the primary care setting are unlikely to be effective without also expanding access to prescribing support for PCPs across the career course.

## Data Availability

All data are available upon reasonable request.
